# Supplementary material for: WordCommentsAnalyzer: A windows software tool for qualitative research
Source: F1000Res. 2018 Sep 4;7:536. Originally published 2018 May 3. [Version 2] doi: 10.12688/f1000research.14819.2 (PMC6137411; doi:10.12688/f1000research.14819.2)
Supplement: Supplementary file 3 [file f1000research-7-17614-s0002.tgz › b2a79496-4ada-47c4-9682-a99ce867836d.zip!/Eight Iranian Health Journals/Journal of Health System Research _36.docx]

© Journal of Health System Research . This work is licensed under CC BY-NC 4.0 (https://creativecommons.org/licenses/by-nc/4.0/).

Volume & Issue: دوره 8، شماره 3؛ 1391

Title: مروری بر ويژگي‌هاي غذا در صنايع تبديلي ارگانيك

http://hsr.mui.ac.ir/index.php/jhsr/article/view/711

صنايع غذايي ارگانيك بخش كوچك ولي پرسرعت در صنايع تبديلي كشورهاي پيشرفته است. محصولات صنايع تبديلي بر حسب نسبت مواد اوليه ارگانيك به كار رفته در فرمولاسيون، تقسيم‌بندي مي‌شوند. استفاده از نگهدارنده‌ها، رنگ‌ها و طعم‌هاي مصنوعی ممنوع است و مواد غير ارگانيك مورد استفاده بايد در استاندارد ارگانيك مجاز شناخته شده باشند. در سال‌هاي اخیر احتمال بالاتر بودن تركيبات زيست فعال مانند ويتامين‌هاي آنتي اكسيدان و تركيبات فنوليك در محصولات ارگانيك قوت بيشتري گرفته است. در شرايط يكسان، محصولات ارگانيك حاوي نيترات و باقي‌مانده‌هاي شيميايي كمتري هستند، ولي محدوديت استفاده از قارچ‌كش‌ها احتمال وجود سموم قارچي را در آن‌ها افزايش مي‌دهد. مقاله حاضر مروري بر ويژگي‌هاي غذاهاي ارگانيک در پنج بخش قوانین، بازار، تولید، ويژگي‌هاي تغذيه‌اي و ايمني غذايي است.

Title: مبانی فقهی جراحي بکارت

http://hsr.mui.ac.ir/index.php/jhsr/article/view/716

محافظت از پرده بکارت ارتباط وثيق به احکام شرعي دارد، شريعت بر محافظت از ناموس و فضيلت و عفت و طهارت تأکيد نموده و در فقه مجازات و ضماني بر عهده ازاله کننده آن قرار داده است. این مطالعه با هدف، مرور منابع فقهی ترمیم بکارت انجام شد. این پژوهش مبتنی بر رویکرد نظری بود و با مطالعه اسناد موجود در این زمینه و جستجوهای انجام گرفته از منابع اطلاعاتی بر روی واژه‌های پرده بکارت و ترمیم بکارت در فقه صورت گرفت. جایز بودن ترميم بکارت در صورتي که تجاوز به عنف و اکراه سبب ازاله پرده بکارت باشد و يا در اثر به زمين خوردن دختر، تصادف و پرش و در صورتي که سبب از بين رفتن پرده بکارت وطئ بر اساس ازدواج باشد ترميم حرام است، خواه بيوه و مطلقه باشد يا نباشد و در صورتي که سبب ازاله بکارت مرتکب شدن به زنا با رضايت باشد و مشهور به زنا نیز باشد ترميم حرام است و اگر مشهور به زنا نباشد و توبه نکرده باشد ترميم جايز نيست، اما اگر توبه کرده باشد، دو احتمال در آن وجود دارد و با توجه به اين که مفسده فريب دادن شوهر در هر حال باقي است، لذا احتمال جايز نبودن ترميم قوي‌تر است. با توجه به اهمیت موضوع، رجوع به منابع فقهی در اقدام بر جراحی ترمیمی بکارت الزامی است.

Title: بررسی ارتباط سرطان پستان با شایع‌ترین عوامل خطر بیماری‌های غیر واگیر در یک مطالعه اکولوژیک

http://hsr.mui.ac.ir/index.php/jhsr/article/view/285

مقدمه: سرطان پستان شایع‌ترین عامل مرگ مرتبط با سرطان در زنان دنیا است؛ به طوری که سالیانه بیش از 1/1 میلیون مورد جدید سرطان پستان در جمعیت زنان جهان رخ می‌دهد. با توجه به اهمیت موضوع، این مطالعه با هدف، تعیین نقش فاكتورهاي غذايي از قبيل مصرف سبزيجات و ميوه‌جات، لبنيات، غذاهاي سرخ كردني، ماهي، نمك به همراه غذا و عواملي هم چون وزن بدن، فعاليت فيزيكي، پرفشاري خون، ديابت، دخانيات، سطح تحصيلات و وضعيت تأهل در ايجاد سرطان پستان انجام گرفت. روش‌ها: ابتدا ضریب همبستگی Pearson دو طرفه بین ميانگين بروز سرطان پستان در سال‌های 85-1380 و تناسب عوامل خطر شایع بیماری‌های غیر واگیر محاسبه شد. عوامل خطری که ضریب همبستگی آن‌ها از لحاظ آماری معنی‌دار بود، جدا گردیده و وارد مدل رگرسیون خطی چند گانه شدند تا ارتباط آن عامل، در طی این سال‌ها هم به صورت انفرادی و هم در صورت وجود عوامل مؤثر دیگر بررسی شوند. یافته‌ها: بر اساس آمارهای موجود، سرطان پستان شایع‌ترین سرطان در زنان ایران و در مجموع سومین سرطان شایع کشور می‌باشد. بر اساس ضریب همبستگی Pearson، بین بروز سرطان پستان در زنان با ابتلا به دیابت، مصرف ماهی در هفته، تحصیلات دانشگاهی و عدم مصرف میوه‌جات در هفته ارتباط مثبت معنی‌دار وجود داشت، ولی بین بروز سرطان پستان با عدم مصرف ماهی در هفته، بی‌سوادی و مصرف میوه‌جات در هفته ارتباط منفی معنی‌دار وجود داشت. Regression analysis نشان داد که تحصیلات دانشگاهی، درصد مصرف میوه‌جات در هفته و ابتلا به دیابت در زنان، دارای ارتباط معنی‌دار بودند. نتیجه‌گیری: از آن جایی که این مطالعه از نوع اکولوژیک مي‌باشد، در تفسیر نتایج آن باید مواظب مغالطه اکولوژیک بود. این مطالعه فقط می‌تواند فرضیه‌هایی را درباره عوامل احتمالی سرطان پستان در ایران به وجود آورد که اثبات آن مستلزم انجام مطالعات دیگری از قبیل هم‌گروهی و موردی- شاهدی می‌باشد.

Title: بررسی میزان آلودگی صوتی در بخش‌های بیمارستان فیض و محوطه اطراف آن

http://hsr.mui.ac.ir/index.php/jhsr/article/view/296

مقدمه: توجه به آرامش و آسایش بیماران در زمان اقامت و بستری در بیمارستان با توجه به وضعیت ناخوشایند آنان، حایز اهمیت می‌باشد. آلودگی صوتی بالاتر از حد مجاز، بیماری این افراد را تشدید می‌کند. همچنین بالا بودن این عامل زیان‌آور روی کارکنان بیمارستان تأثیر می‌گذارد و در نتیجه در ارایه خدمات مناسب به بیماران اثر منفی خواهد گذاشت. بنابراین این تحقیق با هدف بررسی میزان آلودگی صوتی در بخش‌های مختلف بیمارستان فیض و محوطه اطراف آن صورت گرفت. روش‌ها: این مطالعه از نوع توصیفی مقطعی بود که در سه ماه اول سال 1390 به انجام رسید. جهت سنجش میزان صدا در بخش‌های مختلف بیمارستان و خیابان‌های اطراف آن از دستگاه صداسنج مدل 440-CEL استفاده شد. پارامترهای محیطی صدا مانند 10L، 50L، 95L، 95L، 99L، Leq،SEL ،SPL ،Lmin  و Lmax اندازه‌گیری گردید. اندازه‌گیری‌ها در نوبت صبح ساعت 8 تا 12 و بعد از ظهر ساعت 16 تا 20 انجام گرفت. یافته‌ها: حداکثر میانگین تراز معادل صدا (Leq) در نوبت صبح مربوط به بخش درمانگاه 72/66 دسی‌بل و در نوبت بعد از ظهر مربوط به بخش اداری بیمارستان 80/65 دسی‌بل بود. میانگین پارامترهای Lmax، Leq، SEL و 10L در نوبت صبح به ترتیب معادل 87/61، 17/58، 50/61 و 62/60 دسی‌بل و در نوبت بعد از ظهر به ترتیب معادل 67/69، 06/62، 58/71 و 87/64 دسی‌بل بود. نتیجه‌گیری: آزمون‌های آماری نشان داد که بین مقادیر اندازه‌گیری شده صبح و بعد از ظهر اختلاف معنی‌داری وجود داشت (001/0 > P)؛ یعنی میانگین مقادیر شاخص‌های مذکور در نوبت بعد از ظهر بیشتر از نوبت صبح بود. در مجموع مشخص شد که بیمارستان فیض از آلودگی صوتی بالایی برخوردار است و در اکثر موارد، مقادیر بالاتر از استانداردهای سازمان حفاظت محیط‌زیست ایران می‌باشد.

Title: اختلالات اسکلتی- عضلانی در پرسنل پرستاری دارای وظیفه جابجایی بیمار

http://hsr.mui.ac.ir/index.php/jhsr/article/view/295

مقدمه: شغل پرستاری به دلیل ماهیت وظایف از جمله مشاغلی است که در آن WMSDs (Work-related musculoskeletal disorders) از شیوع بالایی برخوردار است. این مطالعه با هدف تعیین شیوع اختلالات اسکلتی- عضلانی و پارامترهای مرتبط با آن در میان پرستاران دارای وظایف جابجایی بیمار صورت گرفت. روش‌ها: در این مطالعه مقطعی که در تابستان 1390 انجام شد، 400 پرستار شاغل در 75 بخش از 11 بیمارستان وابسته به دانشگاه علوم پزشکی شیراز مورد بررسی قرار گرفتند. داده‌ها با استفاده از پرسش‌نامه نوردیک جمع‌آوری شد. داده‌های مطالعه با استفاده از نرم‌افزار آماری 16SPSS تجزیه و تحلیل شد. شیوع اختلالات اسکلتی- عضلانی و فاصله اطمینان 95 درصد محاسبه شد. برای تعیین عوامل مرتبط با اختلالات مقدار P کمتر از 05/0 به عنوان سطح معنی‌داری در نظر گرفته شد. یافته‌ها: میانگین سن و سابقه کار افراد مورد مطالعه به ترتیب 44/6 ± 78/30 و 75/5 ± 92/6 سال به دست آمد. 2/88 درصد از پرستاران مورد مطالعه (2/91 - 2/85؛ 95 درصدCI) در طی 12 ماه گذشته حداقل در یک ناحیه از بدن دچار اختلالات اسکلتی- عضلانی بودند. بیشترین اختلالات در ناحیه کمر (5/71 درصد)، پاها (2/68 درصد)، دست و مچ دست (5/64 درصد)، گردن (2/42 درصد) و شانه (42 درصد) گزارش شد. آنالیز Logistic regression نشان داد که علایم اسکلتی- عضلانی در پرسنل پرستاری مورد مطالعه با سابقه کار، نسبت پرستار به تخت، جنسیت و نوع نظام نوبت‌کاری دارای ارتباط معنی‌دار است (05/0 > P). نتیجه‌گیری: بر اساس یافته‌های مطالعه می‌توان نتیجه گرفت که شیوع WMSDs در میان پرسنل پرستاری دارای وظایف جابجایی بیمار بالا می‌باشد. بنابراین بر اساس این نتیجه و مدل‌سازی رگرسیونی، لازم است عوامل مؤثر در وقوع این اختلالات حذف یا اصلاح شود. همچنین روش‌های جابجایی بیمار بهبود یابد و از وسایل کمکی جابجایی بیمار استفاده شود.

Title: بررسی وضعیت مديريت مواد زاید شهری در مسجد سليمان در ارتباط با سلامت محیط

http://hsr.mui.ac.ir/index.php/jhsr/article/view/464

مقدمه: مدیریت مواد زاید جامد امروزه یکی از مهم‌ترین دغدغه‌های جوامع بشری می‌باشد. در ایران مديريت و ساماندهي مواد زاید در بسیاری از شهرها هيچ گونه مطابقتي با استانداردهاي معمول محيط زيست ندارد و سلامت مردم در معرض خطر می‌باشد. اين تحقيق با هدف، بررسي وضعيت مديريت مواد زاید در مسجد سليمان و تعیین کیفیت شیمیایی شیرابه زباله تولیدی و اثرات ناشی از آن بر سلامت محیط انجام شد. روش‌ها: ابتدا چگونگي منطقه‌بندي شهري و تجهيزات و امكانات شهرداري، منابع توليد زباله، نحوه جمع‌آوري و دفع آن بررسي و سپس مقدار زباله توليدي در ايستگاه‌هاي موقت در هر روز تعيين و مشكلات موجود در سيستم مديريت مواد زاید از توليد تا دفع نهایي مشخص گرديد. نمونه‌ها بر اساس روش استاندارد تهیه و تركيب درصد و رطوبت زباله محاسبه شد. تركيب شيميایي شيرابه زباله از طريق تعيين پارامترهايي مانند COD، BOD، pH، مواد فرار، باقي‌مانده خشك و فلزات سنگين تعيين گرديد.  یافته‌ها: نتايج نشان دادند كه با توجه به ميانگين كل توليد زباله و مقدار سرانه آن در سال به ترتیب برابر 14407663 و 162 کیلوگرم، در سيستم مديريتي موجود مشكلات مختلفي وجود دارد كه سبب انباشته شدن زباله‌ها در نقاط مختلف شهر، نشت شيرابه و تجمع حيوانات ولگرد و حشرات موذي، افزایش بیماری‌های مرتبط و غير بهداشتي شدن فضاي كلي شهر شده است. همچنین مشخص گردید که میانگین غلظت فلزات سنگین Cu، Pb، Ni، Cd، Cr و Zn در شیرابه محل دفع نهایی به ترتیب برابر 528/0، 34/5، 95/3، 309/1، 015/2 و 565/6 و میانگین غلظت COD برابر 2/41523 و BOD برابر 35500 میلی‌گرم در لیتر می‌باشد.

Title: بررسی تأثیر دریافت سویا بر سطح لپتین سرم در یک کارآزمایی بالینی متقاطع در زنان مبتلا به سندرم متابولیک

http://hsr.mui.ac.ir/index.php/jhsr/article/view/709

مقدمه: ممکن است مصرف سویا بر سطح سرمی لپتین مؤثر باشد. هدف از این مطالعه بررسی اثر مصرف سویا بر سطح لپتین سرم در زنان یائسه مبتلا به سندرم متابولیک است. روش‌ها: در این کارآزمایی بالینی متقاطع 42 زن مبتلا به سندرم متابولیک شرکت کردند. سپس افراد به یکی از گروه‌های زیر به مدت 8 هفته تقسیم شدند. گروه شاهد که رژیم غذایی DASH (Dietary approaches to stop hypertension)، گروهی که رژیم غذایی پروتئین سویا و گروهی که رژیم غذایی دانه سویا دریافت کردند. سطح لپتین سرم به روش ELISA (Enzyme-linked immunosorbent assay) اندازه‌گیری شد. یافته‌ها: در طول سه مرحله مداخله تغییر معنی‌داری در وزن دیده نشد. همچنین تفاوت معنی‌داری بین مقادیر نهایی غلظت لپتین سرم بعد از استفاده از این رژیم‌های غذایی مشاهده نگردید (میانگین هندسی ± انحراف معیار: ng/mL 5/2 ± 9/16 در گروه شاهد، ng/mL 6/1 ± 1/16 در گروه پروتئین سویا و ng/mL 7/1 ± 9/15 در گروه دانه سویا). مقایسه درصد تغییرات سطح سرمی لپتین در گروه پروتئین سویا و دانه سویا نسبت به گروه شاهد نشان داد که رژیم‌های حاوی سویا اثری در تغییرات سطح لپتین نداشتند. نتیجه‌گیری: یافته‌های مطالعه حاضر نشان داد که پروتئین و دانه سویا تأثیری بر وزن و سطح لپتین سرم در زنان یائسه مبتلا به سندرم متابولیک ندارد.

Title: بررسی دانش، نگرش و عملکرد پرسنل یک بیمارستان دانشگاهی پیرامون پیش‌گیری از ابتلا به آنفلوانزای نوع H1N1

http://hsr.mui.ac.ir/index.php/jhsr/article/view/712

مقدمه: اقدامات پیش‌گیری از ابتلا به آنفلوانزای نوع H1N1 از اهمیتی حیاتی برخوردار می‌باشد. مطالعه حاضر با هدف، بررسی دانش، نگرش و عملکرد کارکنان درمانی و خدماتی یک بیمارستان دانشگاهی، پیرامون پیش‌گیری از ابتلا به این بیماری صورت گرفت. روش‌ها: اين مطالعه مقطعی در سال 1389 انجام شد و جامعه پژوهش را کلیه کارکنان بیمارستان علی‌ابن‌ابیطالب (ع) رفسنجان تشکیل می‌داد. ابزار جمع‌آوری اطلاعات، پرسش‌نامه محقق ساخته‌ای مشتمل بر 38 سؤال جهت بررسی اطلاعات دموگرافیک (8 سؤال)، دانش (4 سؤال)، نگرش (9 سؤال) و عملکرد (17 سؤال) بود. اطلاعات موجود توسط نرم‌افزار آماری 17SPSS و با استفاده از آزمون 2c مورد تجزيه و تحليل قرار گرفت. یافته‌ها: در این مطالعه 136 نفر از کارکنان اقدام به تکمیل پرسش‌نامه نمودند. دانش و عملکرد پرسنل در سطح خوب و قابل‌قبولی ارزیابی شد و اکثریت آن‌ها نگرش مثبتی نسبت به درمان و پیش‌گیری از آنفلوانزای نوع H1N1 داشتند. نتیجه‌گیری: داشتن دانش خوب، نگرش مثبت و عملکرد مناسب توسط پرسنل، چشم‌انداز روشنی را در رابطه با پیش‌گیری از آنفلوانزای نوع H1N1 نمایان می‌سازد. با این وجود، تعمیم نتایج مطالعه حاضر به دیگر بیمارستان‌های دانشگاهی نیازمند انجام مطالعات بیشتر است.

Title: نگرش دانشجویان دختر دانشکده بهداشت دانشگاه علوم پزشکی اصفهان نسبت به حجاب و پوشش زنان و برخی عوامل مؤثر بر آن

http://hsr.mui.ac.ir/index.php/jhsr/article/view/713

مقدمه: حفظ ارزش‌ها و شعایر اسلامی از وظایف مسلم همه نهادها می‌باشد و رعایت پوشش اسلامی توسط دانشجویان دانشگاه از مصادیق بارز آن به شمار می‌آید. این بررسی با هدف تعیین دیدگاه دانشجویان دختر دانشکده بهداشت در مورد حجاب و برخی از عوامل مؤثر بر آن انجام شد. روش‌ها: مطالعه توصیفی- مقطعی حاضر بر روی 315 دانشجوی دختر دانشکده بهداشت دانشگاه علوم پزشکی اصفهان انجام شد. ابزار مورد استفاده پرسش‌نامه محقق ساخته بود. اطلاعات جمع‌آوری شده در نرم‌افزار SPSS و با استفاده از روش‌های آماری توزیع فراوانی، درصد، میانگین، انحراف معیار و آزمون 2c مورد تجزیه و تحلیل قرار گرفت. یافته‌ها: در زمینه نگرش دانشجویان به حجاب، 4/19 درصد با حجاب موافق، 19 درصد مخالف و 6/61 درصد نظر بینابینی داشتند. بررسی توزیع فراوانی دانشجویان از نظر نوع حجاب در دانشگاه نشان داد که 9/48 درصد از دانشجویان در داخل دانشگاه با چادر، روپوش و مقنعه بودند. بین متغیرهایی مانند رشته تحصیلی دانشجویان (21/0 = P)، تعداد واحدهای گذرانده شده (26/0 = P) و وضعیت تأهل دانشجویان (95/0 = P) با نگرش آن‌ها نسبت به حجاب و پوشش رابطه آماری معنی‌داری دیده نشد؛ در حالی که رابطه بین ترم تحصیلی (031/0 = P) و وضعیت سکونت دانشجویان (008/0 = P) با نگرش آن‌ها به حجاب معنی‌دار بود. نتیجه‌گیری: بر خلاف وجود برخی از دیدگاه‌ها، نتایج به دست آمده بیانگر گرایش به معنویت و ارزش‌های دینی در بین کلیه دانشجویان بود و این گرایش فرصت خوبی را برای مسؤولین فرهنگی دانشگاه فراهم می‌کند که از آن به خوبی و به طور صحیح در جهت ارتقای ارزش‌های اسلامی در بین دانشجویان بهره‌برداری نمایند.

Title: بررسی کیفیت میکروبی و شیمیایی آب آشامیدنی مناطق روستایی بخش مرکزی شهرستان بویراحمد

http://hsr.mui.ac.ir/index.php/jhsr/article/view/714

مقدمه: آب از نخستین نیازهای زندگی و یکی از مفیدترین مواد طبیعت می‌باشد که برای زندگی انسان و تمدن او حیاتی است؛ لذا آگاهی از کیفیت آن برای هر مصرف کننده الزامی می‌باشد. هدف از مطالعه حاضر، تعیین کیفیت میکروبی و شیمیایی آب‌های آشامیدنی مناطق روستایی بخش مرکزی شهرستان بویراحمد بود. روش‌ها: مطالعه توصیفی- مقطعی حاضر در مناطق روستایی بخش مرکزی شهرستان بویراحمد و در سال 1389 انجام شد. در این تحقیق، 79 نمونه برای بررسی کیفیت میکروبی و 21 نمونه برای بررسی کیفیت شیمیایی مورد آزمایش قرار گرفت و نتایج به دست آمده با استفاده از نرم‌افزار 12SPSS تحلیل گردید. یافته‌ها: بر اساس آنالیز آزمایش‌ها مشخص گردید که در 100 درصد موارد، میانگین غلظت پارامترهای شیمیایی) به جز کلر باقی‌مانده و فلوراید) و میکروبی (فیکال کلیفرم) اندازه‌گیری شده در محدوه استاندارد، ولی 24 درصد از موارد میکروبی انداز‌ه‌گیری شده حاوی کلیفرم بود. نتیجه‌گیری: با توجه به این که کمیت و کیفیت قابل قبول آب آشامیدنی مسأله بسیار مهمی است که مردم با آن روبرو هستند و از طرف دیگر، با افزایش جمعیت و منابع آلاینده مانند صنایع و کشاورزی و گذشت زمان به مشکل جدی‌تری مبدل می‌گردد؛ لازم است به طور اساسی به آن پرداخته شود. با توجه به این که بیشترین میزان مغایرت در میزان کلرزنی آب آشامیدنی است، لذا می‌تواند نشانگر نامناسب بودن مدیریت آبداران و بی‌توجهی آن‌ها در امر کلرزنی باشد. در ضمن، بهسازی منابع آب جهت جلوگیری آلودگی آب به سایر کلیفرم‌ها و رعایت استانداردهای آب آشامیدنی جهت بهره‌برداری از منابع آب ضروری است.

Title: تجارب دانشجویان پرستاری از کارورزی با برنامه Mentorship: یک مطالعه کیفی

http://hsr.mui.ac.ir/index.php/jhsr/article/view/422

مقدمه: آموزش باليني، بخشی اساسي از دوره آموزش پرستاري می‌باشد و متأسفانه هنوز يك برنامه آموزشی مناسب كه انتظارات دانشجويان پرستاري را برآورده نمايد، تنظيم و اجرا نشده است. این مطالعه به صورت کیفی و با هدف، تعیین تجارب دانشجویان پرستاری از کارورزی با برنامه منتورشیپ (Mentorship) اجرا گردید. روش‌ها: مطالعه حاضر يك تحقيق كيفي و از نوع آناليز درون مايه‌اي بود که جامعه آماری آن را 16 نفر از دانشجویان ترم آخر دانشکده پرستاری دانشگاه خرم آباد تشکیل می‌دادند. این دانشجویان در نیم‌سال دوم سال تحصیلی 90-1389 تحصیل می‌کردند و کارورزی خود را با برنامه Mentorship گذرانده بودند. براي جمع‌آوري داده‌ها از مصاحبه عميق و نيمه ساختاردار استفاده شد. مدت مصاحبه از 30 تا 45 دقيقه متغير بود و مصاحبه‌ها تا رسيدن به اشباع اطلاعاتي ادامه يافت. سرانجام داده‌ها با کدگذاری در سه سطح یک، دو و سه مورد تجزیه و تحلیل قرار گرفت. یافته‌ها: با بررسی مصاحبه‌ها، 59 کد مفهوم اولیه به دست آمد و با توجه به ماهیت مفاهیم به دو طبقه مزایا و معایب تقسیم شدند. از پنج درون‌مایه فرعی در طبقه مزایا، دو مورد (مشارکت در مراقبت و بهبود کیفیت) تحت عوامل مربوط به آموزش و 3 مورد (رضایت دانشجو، توان و مهارت دانشجو و توان مربی) تحت عوامل فردی قرار گرفت. در طبقه معایب برنامه، از شش درون‌مایه فرعی، دو مورد (پرسنل و مربی) تحت عوامل مدیریت و سه مورد (انضباط آموزشی، طرح درس و محدودیت وقت) تحت عوامل آموزشی قرار گرفت. نتیجه‌گیری: همان طور که از نظرات مشخص است، این برنامه دارای نقاط قوت و ضعفی بود. طبقاتي كه از داده‌هاي اين مطالعه ايجاد شد همگي در راستاي ايجاد ساختار آموزشي مناسب براي يادگيري مطلوب دانشجويان و بهبود ارایه خدمات به جامعه قابل استفاده بود.

Title: بررسی مقدار آلومینیوم باقی‌مانده ناشی از انعقاد متداول و پیشرفته با استفاده از پلی‌آلومینیوم كلراید (PACL) در آب تصفیه شده

http://hsr.mui.ac.ir/index.php/jhsr/article/view/715

مقدمه: پلی‌آلومینیوم كلراید (PACL) یک منعقد کننده معدنی می‌باشد که در سال‌های اخیر کاربرد زیادی جهت حذف رنگ و كدورت در صنعت تصفیه آب پیدا کرده است. در اثر اضافه نمودن مواد منعقد کننده به آب، مقادیری از این مواد در آب حل شده و در آب تصفیه شده نهایی باقی می‌مانند. آلومینیوم یکی از موادی است که در منعقد کننده‌هایی با پایه آلومینیوم (مثل آلوم و PACL) وجود دارد و مقادیر ناچیزی از آن در آب، باعث بروز مشکلات بهداشتی زیادی مانند آلزایمر و آنسفالوپاتی دیالیزی می‌شود. آژانس حفاظت محيط زيست (EPA) حداكثر غلظت مجاز آلومينيوم در آب آشاميدني را mg/l 2/0 تعيين كرده است. انعقاد پيشرفته به صورت اضافه نمودن ماده منعقد کننده اضافی در جهت حذف مواد آلی طبيعي (Natural organic matter یا NOM)، به عنوان پيش‌سازهاي محصولات جانبي گندزدايي (DBPs) تعریف می‌شود و به عنوان بهترین تکنولوژی در دسترس جهت تصفیه و حذف این مواد استفاده می‌گردد. روش‌ها: اين مطالعه بر روي تصفيه‌خانه آب اصفهان انجام شد و مقدار آلومينيوم باقي‌مانده در اثر اضافه نمودن PACL در انعقاد متداول و پيشرفته در مقياس آزمايشگاهي جار تست مورد بررسي قرار گرفت. يافته‌ها: يافته‌ها نشان داد كه ميانگين آلومينيوم باقي‌مانده در انعقاد پيشرفته بيشتر از انعقاد متداول مي‌باشد، ولي مقدار آلومينيوم باقي‌مانده در اين دو مرحله كمتر از حداكثر مجاز توصيه شده توسط EPA مي‌باشد. نتیجه‌گیری: با به كارگيري فرايند انعقاد پيشرفته به همراه استفاده از PACL، نه تنها اهداف اصلي اين فرايند يعني بالا بردن درصد حذف مواد آلي طبيعي و كدورت، محقق گرديد؛ بلكه با چندين برابر كردن مصرف ماده منعقد كننده، مقدار آلومينيوم باقي‌مانده آن كمتر از حداكثر مجاز توصيه شده بود.

Title: عوامل مؤثر بر تفاوت رفتار باروری عشایر اسکان‌یافته و کوچ‌رو سمیرم

http://hsr.mui.ac.ir/index.php/jhsr/article/view/309

از مهم‌ترین مقوله‌های علم جمعیت، مطالعه باروری است. باروری یکی از رویدادهای طبیعی جمعیت و از عناصر مهم رشد جمعیت است. باروری در ظاهر نوعی رفتار بدون مداخله است ولی در عمل متأثر از ارزش‌ها و هنجارهای اجتماعی است که فرهنگ و اعتقادات سنتی افراد نقش مهمی در این امر دارد. این مطالعه با هدف مقایسه بین رفتار باروری عشایر کوچ‌رو و اسکان‌یافته در منطقه سمیرم و یافتن عوامل مؤثر بر این رفتار انجام گردید. روش‌ها در این مطالعه توصیفی و پیمایشی، 357 خانوار از عشایر شهرستان سمیرم با نسبت 70 درصد عشایر کوچ‌رو و 30 درصد اسکان‌یافته به روش نمونه‌گیری تصادفی ساده مورد بررسی قرار گرفتند. ابزار جمع‌آوری، پرسش‌نامه‌ای شامل باورهای سنتی، نگرش به فرزند، نگرش به تنظیم خانواده، مشارکت مردان در تنظیم خانواده، سن ازدواج و تحصیلات بود. داده‌های به دست آمده با کمک نرم‌افزار SPSS با استفاده از روش‌های آمار توصیفی و آمار استنباطی توسط ضریب همبستگی Pearson، آزمون Independent t، 2c و تحلیل رگرسیون پیش‌بینی باروری تجزیه و تحلیل شد. یافته‌ها باورهای سنتی پاسخگویان در خصوص باروری عشایر اسکان‌یافته به طور معنی‌داری بیشتر از عشایر کوچ‌رو است (0001/0 = P). نگرش نسبت به فرزند و تنظیم خانواده، میزان مشارکت مردان در امر تنظیم خانواده، سن ازدواج دختران و سطح تحصیلات در عشایر اسکان‌یافته به طور معنی‌داری بیشتر از عشایر کوچ‌رو است (0001/0 = P). سطح باروری عشایر اسکان‌یافته به طور معنی‌داری کمتر از عشایر کوچ‌رو است (0001/0 = P). نتایج تحلیل رگرسیون نشان داد که فقط باورهای سنتی عشایر در خصوص باروری پیش‌بینی کننده رفتار باروری آن‌ها می‌باشد. بحث اسکان عشایر موجب کاهش باورهای سنتی عشایر در خصوص باروری، تغییر نگرش آنان نسبت به فرزند، امر تنظیم خانواده، مشارکت بیشتر مردان در امر تنظیم خانواده، افزایش سطح تحصیلات، کاهش باروری و نیز افزایش سن ازدواج دختران عشایر شده است. افزایش تحصیلات عشایر نیز می‌تواند منجر به کاهش باروری عشایر گردد.

Title: بررسي ارتباط مصرف غذاهاي آماده Fast food)) با نمايه توده بدني و دور كمر در دختران نوجوان اصفهاني

http://hsr.mui.ac.ir/index.php/jhsr/article/view/717

مقدمه: با توجه به شيوع بالاي چاقي و چاقي شکمي در نوجوانان و ارتباط آن با دريافت غذايي، هدف از اين مطالعه بررسی ارتباط ميان مصرف غذاهاي آماده به عنوان نمايانگري از رژيم غذايي با نمايه توده بدني و دور كمر در دختران نوجوان مقطع راهنمايي مي‌باشد. روش‌ها: اين تحقيق يك پژوهش مقطعي بود كه روي 140 دختر نوجوان 10 تا 14 ساله در مدرسه راهنمايي زينب منطقه جي اصفهان انجام گرفت. دانش‌آموزان به روش تصادفي انتخاب شدند و از آن‌ها فرم FFQ  فعاليت بدني بررسي شد. BMI (Body mass index) كمتر از صدك 15 استاندارد براي سن و جنس به عنوان "كم وزن"، بين صدك 15 تا كمتر از صدك 85 "وزن طبيعي"، بين صدك 85 تا كمتر از صدك 95 "اضافه وزن" و مساوي يا بيشتر از صدك 95 به عنوان "چاقي" طبقه‌بندي شد. يافته‌ها: نتايج اين مطالعه حاکي از آن است که ميانگين BMI در جمعيت برابر 86/19 و دور کمر برابر 69 است و 7/35 درصد اين نوجوانان يا چاق بودند يا اضافه وزن داشتند. همچنين ميانگين دريافت غذاي آماده برابر 6/174 گرم در روز بود. داده‌ها به دست آمده ارتباط معني‌داري ميان مصرف غذاي آماده با نمايه توده بدن و دور کمر نشان داد (009/0 = P، 02/0 = P). نتيجه‌گيري: ارتباط معني‌داري بين مصرف غذاي آماده با دور کمر و نمايه توده بدني در دختران نوجوان اصفهاني ديده شد. از این رو آموزش‌هاي لازم براي نوجوانان ضروري است.

Title: بررسی غلظت فلزات سنگین در آب ورودی به دستگاه دیالیز بیمارستان‌های استان قم و مقایسه آن با استانداردهای AAMI و EPH

http://hsr.mui.ac.ir/index.php/jhsr/article/view/718

مقدمه: وجود فلزات سنگین در آب ورودی به دستگاه‌های دیالیز و ورود آن به بدن بیماران دیالیزی از طریق همودیالیز (به علت میزان آب زیاد دریافتی در هر بار دیالیز) باعث بروز مشکلات بسیار زیادی از قبیل مسمومیت‌های حاد، بیماری‌های مغزی، استخوانی و غیره می‌شود. بنابراین رعایت استانداردهای مربوطه در زمینه مقدار فلزات سنگین در آب دیالیز ضروری به نظر می‌رسد. این تحقیق در سال 1389 روی نمونه آب ورودی به دستگاه‌های دیالیز بیمارستان‌های استان قم برای مقایسه مقادیر فلزات سنگین موجود در آن‌ها و مقایسه با استانداردهای موجود صورت گرفت. روش‌ها: پژوهش حاضر مطالعه توصیفی تحلیلی است که به صورت مقطعی روی تعداد 45 نمونه از ورودي دستگاه دياليز بيمارستان‌هاي استان قم، صورت گرفت. با توجه به اين كه تعداد 3 بيمارستان در استان قم داراي واحد دياليز می‌باشد، از هر بیمارستان تعداد 15 نمونه برداشت گردید. نمونه‌ها با دستگاه اتمیک مورد آزمایش قرار گرفتند. داده‌ها با استفاده از نرم‌افزار 17SPSS مورد آنالیز قرار گرفتند. یافته‌ها: میانگین و انحراف معیار غلظت آلومینیوم، سرب و کادمیوم به ترتیب برابر 50/4 ± 29/4، 42/0 ± 84/2 و 18/0 ± 53/0 میکروگرم بر لیتر و روی برابر با 01/0 ± 002/0 میلی‌گرم در لیتر به دست آمد که این مقادیر از حد استانداردهای AAMI (Association for the advancement of medical instrumentation) و EPH (Extractable Petroleum Hydrocarbon)  پایین‌تر بود. در تعداد کمی از نمونه‌ها این مقادیر بالاتراز حد استاندارد بود. نتیجه‌گیری: میانگین فلزات سنگین اندازه‌گيري شده در اين بيمارستان‌ها كمتر از حد استاندارد بود. به عبارت ديگر بيماران در شرايط خوبی نسبت به عوارض ناشي از موارد ذكرشده به سر مي‌برند.

Title: پتانسیل خورندگی و رسوب‌گذاری آب آشامیدنی در شبکه توزیع شهر کوهدشت با استفاده از اندیس‌های خورندگی

http://hsr.mui.ac.ir/index.php/jhsr/article/view/719

مقدمه: یکی از عوامل تأثیرگذار در کیفیت آب خورندگی است. عدم كنترل كيفيت شيميايي آب در شبكه توزيع باعث وقوع پديده‌هاي خورندگي و رسوب‌گذاري و در نتیجه آسيب‌هاي اقتصادي و بهداشتي مي‌شود. هدف اين مطالعه بررسي كيفيت شيميايي آب آشاميدني مورد استفاده در شهر كوهدشت و تعين پتانسيل خوردندگي و رسوب‌گذاري آن است. روش‌ها: در این مطالعه توصیفی- مقطعی، به طور منظم از 12 منطقه از پیش تعیین شده شهر کوهدشت در دو مرحله نمونه‌برداری انجام شد. مقادیر اندیس‌های لانژلیه، رایزنر، خورندگی و پوکوریوس با تعیین پارامترهای درجه حرارت، سختی کلسیم، قلیائیت، کل جامدات محلول و pH محاسبه گردید. از آزمون student t براي تحليل داده‌ها و مقايسه ميانگين‌ها استفاده شد. یافته‌ها: مقادیر اندیس‌های لانژلیه 47/0-، رایزنر 86/7، خورندگی 43/11 و پوکوریوس 49/6 به دست آمد. نتیجه‌گیری: نتايج حاكي از اين واقعيت است كه آب شهر کوهدشت دارای خاصیت متمایل به خورندگی است. از این رو مسؤولين آب شهري مي‌بايست اقداماتي در زمينه تعديل pH و تثبيت آب انجام دهند.

Title: بررسی ميزان سرب در مارک‌های پرمصرف محصول آرايشی سايه چشم در شهر اصفهان

http://hsr.mui.ac.ir/index.php/jhsr/article/view/443

مقدمه: سرب از راه‌های مختلف اعم از خوراکی، تنفسی و پوستی وارد بدن انسان می‌گردد. یکی از منابع پوستی ورود سرب به بدن توسط مواد آرایشی مانند رژ لب و سایه چشم می‌باشد. ورود سرب به بدن باعث ایجاد خطراتی مانند فشار خون دوران بارداری، سقط جنین و کاهش عملکرد سیستم عصبی نوزادان می‌گردد. در این مطالعه، میزان سرب در رایج‌ترین سایه چشم‌های مصرفی در بازار مورد بررسی قرار گرفت تا ایمنی و کیفیت آن‌ها سنجیده شده و در نهایت مرغوب‌ترین نوع آن‌ها تعیین گردد. روش‌ها: مطالعه توصیفی- تحلیلی حاضر در سال 1390 در شهر اصفهان انجام شد. اطلاعات مربوط به پرمصرف‌ترین مارک‌های ماده آرایشی سایه چشم از طریق فروشندگان بزرگ محصولات آرایشی به دست آمد. با استفاده از روش‌های آماری، تعداد 15 نمونه پرمصرف از این نوع ماده آرایشی انتخاب و با استفاده از دستگاه جذب اتمی مورد تجزیه و تحلیل قرار گرفت. یافته‌ها: غلظت سرب در نمونه‌های مورد مطالعه بین 6- 85/0 میکروگرم بر گرم بر اساس وزن تر به دست آمد و میانگین کلی میزان سرب در تمام نمونه‌های مورد مطالعه برابر 53/3 میکروگرم بر گرم بود. بیشترین و کمترین غلظت سرب به ترتیب مربوط به مارک‌های فلورمار با میزان 66/5‌ و کاویان با مقدار 5/1 میکروگرم بر گرم بود. نتیجه‌گیری: در این مطالعه غلظت سرب در سایه‌های چشم مورد مطالعه کمتر از مقدار توصیه شده توسط سازمان غذا و داروی آمریکا (Food and drug administration یا FDA) بود. لذا جهت جلوگیری از اثرات مضر بهداشتی به خصوص برای کودکان و زنان باردار، باید همواره اندازه‌گیری مقدار سرب در محصولات آرایشی مورد پایش قرار بگیرد تا از اثرات زیانبار آن کاسته شود.

Title: بررسی میزان وزن‌گیری مادران باردار در دوران بارداری بر اساس استانداردهای توصیه شده و ارتباط آن با وزن هنگام تولد نوزادان در شهر اصفهان

http://hsr.mui.ac.ir/index.php/jhsr/article/view/335

مقدمه: یکی از تغییرات مهم دوران بارداری، افزایش وزن زن باردار است که از شاخص‌های معتبر تغذیه می‌باشد. وزن‌گیری مطلوب طی بارداری به دلیل وجود نظریه‌های متناقض امروز، موضوع بحث و مطالعات متعددی است. با توجه به این که تاکنون در کشورمان مطالعات محدودی در زمینه ارتباط وزن‌گیری مادر باردار در دوران بارداری با وزن هنگام تولد نوزاد انجام شده است، بنابراین این مطالعه با هدف، تعیین میزان وزن‌گیری مادر باردار در دوران حاملگی بر اساس استانداردهای توصیه شده و نیز تعیین ارتباط آن با وزن هنگام تولد نوزادان در شهر اصفهان انجام شد. روش‌ها: پژوهش حاضر یک مطالعه توصیفی- تحلیلی- مقطعی بود و ابزار جمع‌آوری داده‌ها را فرم ثبت اطلاعاتی تشکیل می‌داد که با توجه به داده‌های ثبت شده در پرونده بهداشتی خانوار تکمیل گردید. حجم نمونه مشتمل بر 225 مادر مراجعه کننده به مراکز بهداشتی درمانی شهر اصفهان بود که به روش خوشه‌ای چند مرحله‌ای انتخاب و مورد مطالعه قرار گرفتند. داده‌ها با استفاده از آزمون‌های آماری مناسب و نرم‌افزار SPSS و در سطح معنی‌داری کمتر از 05/0 تجزیه و تحلیل گردید. یافته‌ها: میانگین افزایش وزن دوران بارداری 73/11 کیلوگرم بود. میانگین وزن نوزادان هنگام تولد 3193 گرم به دست آمد که 11/7 درصد آنان کمبود وزن هنگام تولد (Low birth weight یا LBW) و 22/6 درصد وزن بیش از 4000 گرم داشتند. میانگین شاخص توده بدنی (Body mass index یا BMI) گروه تحت مطالعه قبل از بارداری نیز 3/25 کیلوگرم بر مترمربع بود. ضریب همبستگی Pearson ارتباط مستقیم معنی‌داری را بین BMI قبل از بارداری و میزان افزایش وزن دوران بارداری با وزن نوزاد هنگام تولد نشان داد. نتیجه‌گیری: میانگین افزایش وزن در دوران بارداری شرایط به نسبت مطلوبی را در این مطالعه نشان داد، اما در جزییات افزایش وزن در زیر گروه‌های BMI مشخص شد که درصد قابل توجهی از زنان لاغر و نرمال نتوانسته‌اند به میزان توصیه شده افزایش وزن داشته باشند و از سویی دیگر درصد قابل توجهی از زنان دارای اضافه وزن و چاق، افزایش وزن بیش از میزان استاندارد داشتند. با این وجود ارتباط مستقیم معنی‌داری بین افزایش وزن دوران بارداری با وزن هنگام تولد نوزاد وجود داشت.

Title: تجزیه روغن‌های آسکارل (PCB170 و 153 PCB) با استفاده از پرتو میکروویو و تلفیق همزمان TiO2/H2O2

http://hsr.mui.ac.ir/index.php/jhsr/article/view/339

مقدمه: ترکیب‌های بی‌فنیل‌های چند کلره (PCBS) از جمله آلاینده‌های خطرناک شغلی و محیطی است. این مواد جزو يكي از دوازده گروه تركيبات آلی محسوب مي‌گردند که چالش‌های شغلی و زیست‌محیطی عمده‌ای را ایجاد می‌کند. این ترکیب‌ها اثرات بهداشتی مختلفی را با توجه به راه ورود، جنس، سن، غلظت و سطح مواجهه ایجاد می‌کند. هدف از اين مطالعه، تجزیه روغن‌های آسکارل با استفاده از پرتوهای میکروویو و تلفیق  TiO2/H2O2 جهت کاهش مواجهه کارگران می‌باشد. روش‌ها : بررسی حاضر مطالعه تجربی آزمایشگاهی بود که در دانشگاه تربیت‌مدرس در ماه‌های خرداد تا شهریور 1390 انجام شد. راکتور مورد استفاده یک بالن ژوژه پیرکس ته پهن cc 250 بود که به وسیله یک لوله رابط از طریق منفذی که در بالای میکروویو قرار داشت به یک کندانسور ارتباط پیدا می‌کرد. دستگاه میکروویو با فرکانس 2450 مگاهرتز و توان خروجی 900 وات بود. توان پرتو در محدوده 540، 720 و 900 وات مورد استفاده قرار گرفت. pH نمونه‌ها و دما به طور مداوم پایش شد. آزمایش‌ها به صورت سه بار تکرار گردید و نتایج، میانگین سه بار تکرار می‌باشد. روغن‌های آسکارل با استفاده از دستگاه GC-ECD مورد آنالیز قرار گرفت. سپس داده‌ها وارد نرم‌افزار 16SPSS گردید و با استفاده از تحلیل واریانس با اندازه‌گیری‌های مکرر و آزمون آماری t-test تجزیه و تحلیل شد و P کمتر از 05/0 معنی‌دار تلقی گردید. یافته‌ها: میانگین تجزیه برای مجموع PCBها بر حسب توان پرتو میکروویو با 540، 720 و 900 وات به ترتیب 25/84، 81/88 و 84/95 بود. میانگین تجزیه برای مجموع PCB‌ها بر حسب نسبت حلال مورد استفاده به روغن آسکارل به میزان 1:1، 2:1 و 3:1 به ترتیب 82/53، 06/79 و 95/94 بود. میانگین تجزیه بر حسب عدم استفاده و به کارگیری H2O2 به میزان 10 درصد و 20 درصد به ترتیب 9/70، 65/84 و 91/88 بود. میانگین تجزیه بر حسب عدم استفاده و به کارگیری با نسبت 05/0، 10/0، 15/0 و 20/0 گرم به ترتیب 9/70، 53/76، 32/77، 10/79 و 19/80 بود. میانگین تجزیه بر حسب عدم استفاده و به کارگیری همزمان H2O2 به میزان 10 درصد و TiO2 با نسبت 05/0، 10/0، 15/0 و 20/0 گرم به ترتیب 9/70، 3/89، 18/91، 17/92 و 16/93 بود. همچنین میانگین تجزیه بر حسب عدم استفاده و به کارگیری همزمان H2O2 به میزان 20 درصد و TiO2 با نسبت‌های 05/0، 10/0، 15/0 و 20/0 گرم به ترتیب 9/70، 95/93، 95/94، 84/ 95 و 22/97 بود. نتیجه‌گیری: نتايج تحقيق روي تركيب‌های PCB با استفاده از پرتوهاي ميكروويو در حضور حلال اتانول و اكسيدكننده قوي H2O2 و کاتالیزور TiO2 منجر به تجزیه یا کاهش میزان PCB‌ در روغن ترانسفورمر ‌شد. در واقع با افزایش مقدار اتانول میزان تولید رادیکال هیدروکسیل افزایش یافت و موجب کلرزدایی بیشتر مولکول PCB گردید. همچنین میزان تجزیه PCB با افزایش غلظت H2O2 و ازدیاد نسبت  TiO2 افزایش یافت.

Title: ارتباط کیفیت خواب با کیفیت زندگی در بيماران دياليزي مراكز دياليز استان چهارمحال و بختياري

http://hsr.mui.ac.ir/index.php/jhsr/article/view/297

مقدمه: ایجاد نارسایی مزمن کلیه و در نهایت همودیالیز سبب ایجاد عوارضی می‌شود که می‌تواند کیفیت خواب بیمار و ابعاد مختلف کیفیت زندگی او را تحت  تأثیر قرار دهد. از این رو مطالعه حاضر با هدف تعیین ارتباط کیفیت خواب با کیفیت زندگی در بيماران همودياليزی استان چهارمحال و بختياري طراحی شد. روش‌ها: این پژوهش از نوع توصیفی- تحليلي بود. جمعيت مورد مطالعه، بیماران همودیالیزی مراجعه‌کننده به مراکز همودیالیز استان چهارمحال و بختیاری بودند که به صورت سرشماری وارد مطالعه شدند. ابزار جمع‌آوری داده‌ها، پرسش‌نامه با سه بخش مربوط به مشخصات دموگرافیک، پرسش‌نامه کیفیت خواب پیتزبرگ و کیفیت زندگی سازمان بهداشت جهانی بود. اطلاعات با نرم افزار 15SPSS و آزمون‌های آماری Mann-Whitney، 2c و Independent t مورد تجزیه و تحلیل قرار گرفت. یافته‌ها: افراد مورد پژوهش 171 نفر بیمار دیالیزی بودند. جنسيت افراد مورد بررسي 95 نفر مرد (6/55 درصد)، 76 نفر زن (4/44 درصد) بودند. میانگین سن در مردان 48/16 ± 12/59 و در زنان03/18 ± 55/55 بود و اختلاف میانگین سن در دو جنس معنی‌دار نبود (18/0 = P). میانگین مدت زمان انجام دیالیز 03/33 ± 96/36 ماه بود. هیچ کدام از بیماران دیالیزی در دسته بندی با کیفیت زندگی مطلوب قرار نداشتند. همچنین آزمون 2c نشان داد که کیفیت خواب بر کیفیت زندگی بیماران همودیالیزی تأثیر دارد و این ارتباط از نظر آماری معنی‌دار بود (05/0 P <). نتیجه گیری: با توجه به یافته‌های پژوهش حاضر، ضرورت توجه به کیفیت خواب این بیماران به عنوان یکی از ابعاد مهم تأثیرگذار در ارتقای کیفیت زندگی بیش از بیش مطرح می‌گردد.
